# What are the implementation lessons from a high-volume cataract screening and surgical programme? A mixed-methods implementation evaluation in Sierra Leone

**DOI:** 10.1136/bmjopen-2026-126880

**Published:** 2026-10-09

**Authors:** Gabriel Osei-Anokye, Muhammad Rabiu, Lloyd Harrison-Williams, John G Mattia, Ving Fai Chan

**Affiliations:** 1 Centre for Public Health, Queen’s University Belfast, Belfast, UK; 2 Noor Dubai Foundation, Dubai, UAE; 3 Connaught Teaching Hospital, Freetown, Sierra Leone; 4 Choithram Memorial Hospital, Freetown, Sierra Leone; 5 Lowell and Ruth Gess, UMC Memorial Eye Hospital, Freetown, Sierra Leone; 6 College of Health Sciences, University of KwaZulu-Natal, Durban, South Africa

**Keywords:** Cataract, vision-related quality of life, RE-AIM framework, Implementation science, Sierra Leone

## Abstract

**Abstract:**

**Objective(s):**

To evaluate the first implementation cycle of the cataract screening and surgical programme in Sierra Leone using the Reach, Effectiveness, Adoption, Implementation and Maintenance framework and the Plan-Do-Study-Act approach.

**Design:**

Mixed-methods implementation evaluation of routine programme data with interviews and focus group discussions.

**Setting:**

Sierra Leone.

**Participants:**

Cataract-positive patients identified through community screening.

**Primary and secondary outcomes:**

Primary outcome includes programme reach and implementation, vision-related quality of life measured. Secondary outcomes included qualitative assessment of implementation barriers and programme adaptations.

**Results:**

Among 9117 individuals screened, 7738 complete records were analysed, and 1433 cataract surgeries were performed. The programme predominantly reached economically vulnerable populations and mean composite National Eye Institute Visual Function Questionnaire score improved by 17 points following surgery (p<0.001). High patient demand, transportation and road infrastructure barriers, limited workforce and equipment affected implementation and postoperative follow-up. Adaptation included changes to staffing and workflows and plans for decentralised follow-up services.

**Conclusions:**

High-volume cataract outreach in Sierra Leone substantially improved vision-related quality of life while highlighting the importance of adaptive implementation strategies in resource-constrained settings.

**STRENGTHS AND LIMITATIONS OF THIS STUDY**

- The combined use of the Reach, Effectiveness, Adoption, Implementation and Maintenance framework and Plan-Do-Study-Act approach enabled a comprehensive evaluation of the programme implementation.
- The mixed-methods implementation evaluation generated practical insights into delivering high-volume cataract services in real-world settings.
- Use of routine programme data resulted in some missing and incomplete records.
- The evaluation was limited to the first implementation cycle and could not assess long-term sustainability.

## Introduction

 Cataract remains the leading cause of avoidable blindness worldwide, disproportionately affecting populations in low- and middle-income countries (LMICs) where access to timely surgical care is often limited.^1 2^ Although cataract surgery is a highly cost-effective intervention, major gaps in service delivery persist across many LMICs.^3 4^ In countries such as Sierra Leone, systemic constraints including shortages of ophthalmic workforce, inadequate infrastructure, weak transport systems, financial barriers and limited surgical capacity contribute to delayed presentation and a high burden of untreated cataract.^5^ Outreach-based cataract programmes are therefore frequently used to expand access to surgery in settings where routine eye-care services remain insufficient.

While cataract outreach programmes are widely implemented across LMICs, published evaluations have largely focused on surgical output, visual outcomes and service uptake.^6–10^ Comparatively, less attention has been given to the realities of how these programmes are established, operationalised and adapted within complex and resource-constrained health systems, particularly during their early phases of implementation.^8^ Key aspects relating to workforce mobilisation, infrastructure readiness, patient flow, supply-chain management, data systems, cultural negotiation and adaptation to local realities remain poorly documented in the literature.^11^ This represents a critical gap as understanding these operational and contextual dynamics is essential for designing programmes that are not only effective in the short term, but also equitable, sustainable and capable of integration within existing health systems.

The Choithram Memorial Hospital (CMH) cataract outreach programme in Sierra Leone conceptualised by Noor Dubai Foundation was established as a 3-year programme aimed at expanding access to cataract surgery while simultaneously strengthening local delivery systems through workforce development, infrastructure investment and collaborative implementation between local partners like the Ministry of Health in Sierra Leone, the Noor Dubai foundation and Choithram Netralaya Hospital in Indore, India. Importantly, the first implementation cycle functioned not only as a service delivery initiative, but also as a real-world systems-learning phase during which operational challenges, contextual barriers and implementation adaptations emerged in real time.

This study examines the first implementation cycle of a large-scale cataract outreach programme in Sierra Leone as a case of ‘learning while delivering’ within a resource-constrained setting. Using the Reach, Effectiveness, Adoption, Implementation and Maintenance (RE-AIM) framework and Plan-Do-Study-Act (PDSA) approach as complementary frameworks for implementation evaluation and iterative learning, we examined how the programme was established, operationalised and adapted in response to emerging implementation challenges and local realities. By examining processes of adaptation, system constraints and real-time decision-making alongside programme outcomes, the study aims to generate practical implementation learning for strengthening cataract outreach and similar health interventions in comparable low-resource settings.

## Methods

### Study design

This study was conducted as a mixed-methods implementation learning study and programme evaluation of the first implementation cycle of the Choithram Memorial Hospital Cataract Screening and Surgical Programme in Sierra Leone between February and August 2025. This programme formed part of a 3-year cataract outreach initiative designed to expand access to cataract services while strengthening local eye-care capacity and health system integration.

The first implementation cycle functioned as a real-world systems-learning phase aimed not only at delivering high-volume cataract screening and surgery, but also at understanding how the programme operated, adapted and evolved during implementation in response to local realities. The evaluation combined quantitative analysis of routine programme monitoring data with qualitative insights from key stakeholders and programme beneficiaries to provide a comprehensive assessment of programme performance and implementation processes.

### Conceptual framework

This study was guided by the combined application of the RE-AIM framework and the PDSA cycle to support both implementation evaluation and programme learning during the first implementation cycle.^12 13^

The RE-AIM framework was used to structure evaluation across five domains: Reach, Effectiveness, Adoption, Implementation and Maintenance. The framework supported assessment not only of clinical outputs and patient outcomes, but also of programme delivery processes, service utilisation, continuity of care and factors influencing sustainability within the Sierra Leone eye health system.

The PDSA cycle complemented this by providing an iterative learning framework for understanding how programme delivery evolved during implementation. The ‘Plan’ phase involved development of the outreach and surgical delivery model; the ‘Do’ phase involved implementation of screening, referral, surgical and follow-up activities; the ‘Study’ phase involved evaluation of programme performance using quantitative and qualitative findings; and the ‘Act’ phase focused on identifying operational adaptations and refinements to strengthen subsequent implementation cycles.

Together, these frameworks supported examination of both programme outcomes and the adaptive processes through which the outreach model evolved within a resource-constrained setting.

### Study setting and programme context

Sierra Leone faces a substantial burden of avoidable blindness, with cataract as the leading cause of visual impairment among older adults.^5 14^ The programme aimed to screen about 15 000 adults for cataract and provide surgical treatment for at least 6000 eligible patients, with a primary focus on individuals aged 50 years and above, who bear the highest burden of cataract-related blindness.^15^ The first implementation cycle employed a high-volume outreach model combining community-based screening activities with centralised surgical delivery at CMH in Freetown, which served as the tertiary referral and surgical centre.

Community outreach screening camps were conducted in selected locations and provinces with the help of the Ministry of Health to identify individuals with suspected cataract and other eye conditions. Patients identified as requiring surgery were referred through a structured referral pathway to CMH for further assessment and surgical management. Surgical services were delivered through collaboration between visiting ophthalmic teams from Choithram Netralaya Hospital in India. In addition to service delivery, the programme incorporated capacity-building components, including mentorship, training and collaborative working with local healthcare providers to support long-term sustainability and local ownership.

Postoperative follow-up was scheduled at 1 day, 1 week and 1 month following surgery, using a combination of hospital-based review at CMH for patients in Freetown and district hospital reviews for patients in other provinces. As this represented the first implementation cycle, programme systems and operational workflows continued to evolve throughout implementation. Programme delivery was influenced by transportation barriers, infrastructure limitations and seasonal conditions, including the rainy season.

### Description of the first implementation cycle

The first implementation cycle represented the establishment and operational learning phase of a 3-year cataract outreach programme aiming to deliver approximately 6000 cataract surgeries across Sierra Leone (figure 1). The programme was implemented through a partnership between the Sierra Leone Ministry of Health, Noor Dubai Foundation, CMH and Choithram Netralaya Hospital. A decentralised screening and referral model with centralised surgery at CMH was adopted. Ophthalmic Nurses and Ophthalmic Community Health Officers conducted community screening, counselling, referral coordination and postoperative follow-up, while patients with operable cataracts (visual acuity (VA) <6/24) were referred to CMH for surgery. The programme provided transport, accommodation, meals and perioperative care to minimise financial and geographic barriers. Surgical services were delivered by multidisciplinary teams comprising visiting ophthalmologists, CMH personnel and Ministry of Health staff. The evaluation examined programme implementation, including referral pathways, patient flow, staffing, follow-up systems and operational adaptations during the first implementation cycle.

**Figure 1:**
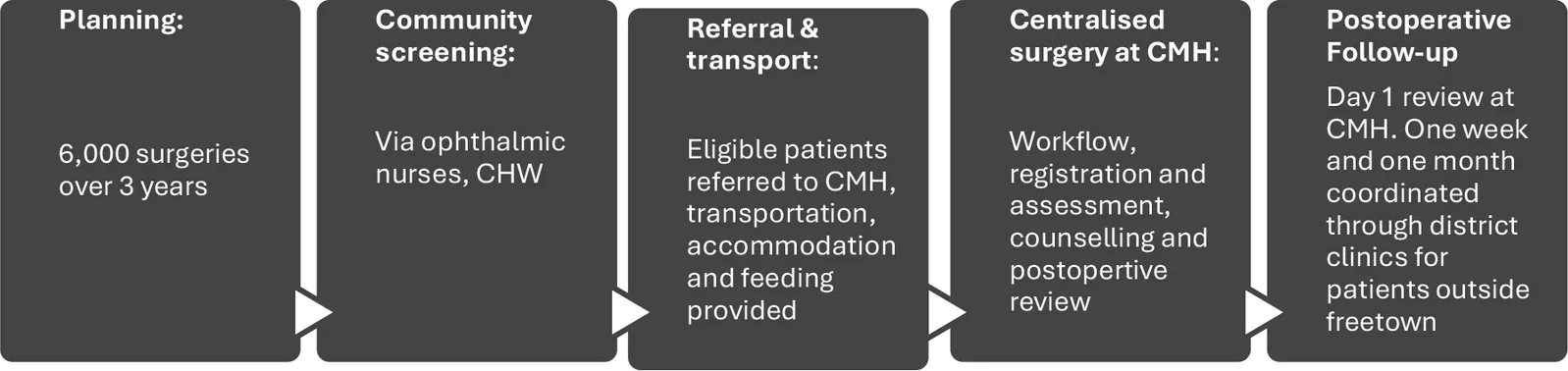
First implementation cycle. Schematic overview of the first implementation cycle, illustrating the pathway from community-based cataract screening and identification of eligible patients through referral, preoperative assessment, cataract surgery and postoperative follow-up. CMH, Choithram Memorial Hospital.

### Quantitative data sources and measures

Quantitative data for this study were obtained from routine programme monitoring and clinical service records. Data sources included community screening registers, referral documentation, surgical logs, postoperative follow-up records and programme monitoring databases maintained at CMH (table 1). Data were collected and managed using Kobo Collect, an open-source electronic data collection platform, which was suitable because of its offline functionality and ability to use in remote settings with limited internet connectivity.

**Table 1:** Quantitative data sources and domain

Domain/indicator	Information collected	Data source	Planned analysis
Participants demographics	Age, sex, district	Screening register	Descriptive statistics
Screening, referral and surgical outcomes	Number screened, VA’s, referral status, surgical uptake	Screening register, referral documentation, surgical logs	Screening coverage, referral uptake proportions, surgical rate
Postoperative follow-ups	Attendance at day 1, 1-week and 1-month follow-up visits	Follow-up register; programme monitoring database	Follow-up attendance rate; attrition analysis
Visual outcomes	Postoperative VA’s	Clinical records;	Visual acuity outcome analysis
Vision-related quality of life	VFQ-25 composite and subscale scores	VFQ-25 questionnaire	Pre and post comparison of QoL scores using paired analysis
Implementation and system learning	Operational adaptation, workflow modification, follow-up challenges	Programme monitoring reports	Framework-informed interpretive analysis guided by RE-AIM and implementation-learning objectives

QoL, quality of life; RE-AIM, Reach, Effectiveness, Adoption, Implementation and Maintenance; VA, visual acuity; VFQ-25, Vision function questionnaire-25.

Quantitative indicators captured demographic characteristics, screening and referral outcomes, surgical output, postoperative follow-up attendance, visual outcomes, vision-related quality of life and operational programme performance. Vision-related quality of life was assessed using the National Eye Institute Visual Function Questionnaire (VFQ-25), administered preoperatively and postoperatively among patients with complete paired data.

### Qualitative data collection

Qualitative data were collected to explore experiences of establishing, delivering and adapting the cataract outreach programme during its first implementation cycle. A qualitative descriptive approach was used to explore participants’ experiences of programme implementation and adaptations made during delivery. Semistructured interviews and two focus group discussions (FGDs) were conducted with a purposive sample of programme stakeholders, including 6 programme implementers, 5 ophthalmologists, 3 ophthalmic nurses, 2 counsellors, 3 operational staff and 32 patients (table 2). Interview and FGD guides were informed by the RE-AIM framework and focused on service delivery, implementation challenges, barriers to care, follow-up and programme adaptations. Each FGD included six participants (three men and three women), representing Freetown and provincial districts. Interviews and FGDs were conducted confidentially, audio-recorded with consent, transcribed verbatim and anonymised before analysis.

**Table 2:** Qualitative data sources and domains for the first-year cycle

Qualitative domain	Participants	Key areas explored	Planned analysis
Programme implementation	Ophthalmologists, optometrists, implementers	Experiences of programme delivery, workflow processes, operational coordination, staffing	Framework-informed interpretive analysis guided by RE-AIM and implementation-learning objectives.
Surgical surgery delivery	Ophthalmologists, ophthalmic nurses, operated patients	Patient experiences, staffing pressure, surgical flow and outcomes	Framework-informed interpretive analysis guided by RE-AIM on services delivery, experiences and systems
Postoperative follow-up pathways	Patients, implementers, ophthalmologists	Follow-up attendance, transportation barriers, caregivers’ dependence, seasonal disruptions	Exploration of structural barriers affecting continuity of care
Patient experiences	Screened patients, failed screening, received surgery, attended follow-up, did not attend follow-up	Perceptions of cataract surgery, fear and misconception, family influence, quality of life changes	Analysis of social, cultural and behavioural influences on programme uptake
Programme adaption and systems learning	Implementers, clinicians and operational staff	Operational learning, workflow modifications, staffing adaptation, data management challenges, sustainability planning	Identification of adaptive implementation processes and systems learning
Sustainability and future programme planning	Programme implementers, local health workers	Long-term sustainability, workforce capacity, decentralisation, health system integration, future redesign	Framework-informed interpretation of sustainability and scale-up considerations

RE-AIM, Reach, Effectiveness, Adoption, Implementation and Maintenance.

## Data analysis

### Quantitative analysis

Quantitative data were analysed descriptively to assess programme performance across the RE-AIM domains. Demographic characteristics, screening outcomes, surgical volume, postoperative VA, follow-up attendance, referral adherence, documentation completeness and implementation metrics were summarised using appropriate descriptive statistics. Continuous variables were reported as means (SD) or medians (IQR), depending on distribution, and age was categorised into predefined programme groups. Vision-related quality of life was assessed using the Wilcoxon signed-rank test to compare paired preoperative and postoperative VFQ-25 scores. Analyses were restricted to participants with complete follow-up data, with no imputation for missing data. No sample size calculation as this was a descriptive implementation evaluation of routine programme data.

### Qualitative analysis

Qualitative data were analysed using a qualitative descriptive, framework-informed interpretive approach. The analysis was situated within a pragmatic orientation, reflecting the study’s aim of generating applied understanding of programme implementation rather than developing an explanatory theory. Two researchers (GO-A and VFC) independently reviewed the interview and focus group transcripts in May 2026. Analysis proceeded through familiarisation with the data, identification of implementation-relevant segments, and organisation of these findings using the RE-AIM domains and the study’s implementation-learning objectives. Within and across these domains, the researchers identified recurring experiences, operational challenges, barriers and facilitators, patient perspectives, and adaptations, while remaining open to findings not readily accommodated within the predefined framework. Interpretations were compared across stakeholder groups and data sources, and differences between researchers were discussed and resolved through consensus. The resulting categories were subsequently integrated with quantitative programme findings to identify areas of convergence, divergence and explanatory complementarity (figure 2).

**Figure 2:**
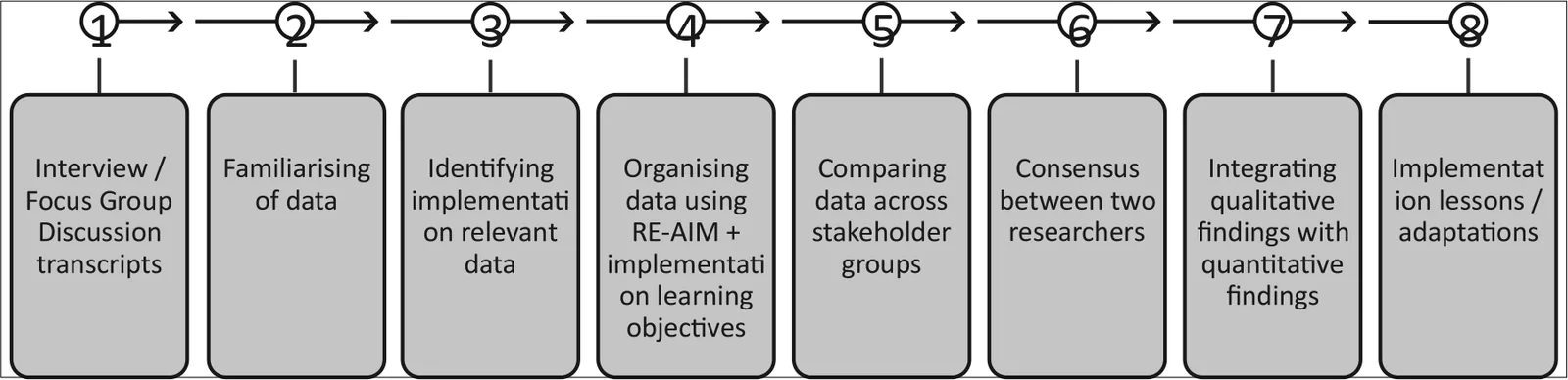
Qualitative data analysis pathway. Overview of the systematic process used to analyse qualitative data from interview and focus group discussion transcripts. The pathway involved data familiarisation, identification of implementation-relevant data, organisation using the RE-AIM framework and implementation learning objectives, comparison across stakeholder groups, consensus between two researchers and integration of qualitative and quantitative findings to generate implementation lessons and adaptations. RE-AIM, Reach, Effectiveness, Adoption, Implementation and Maintenance.

### Integration of quantitative and qualitative findings

Quantitative and qualitative findings were integrated using a triangulation approach to provide a comprehensive understanding of programme performance and implementation processes. Quantitative findings provided measurable indicators of programme reach, surgical outcomes, follow-up attendance and operational performance, while qualitative findings helped explain the underlying mechanisms, implementation experiences and ground realities influencing these outcomes. This integrated analytical approach enabled identification of how programme adaptations emerged in response to challenges and how system-level constraints influenced both implementation processes and observed outcomes. No sensitivity analyses were performed.

### Reflexivity statement

The evaluation team (GO-A and VFC) worked closely with programme implementation, providing contextual insight into operational challenges and service delivery during the first implementation cycle. GO-A contributed to study design, coordination, and interpretation, drawing on experience in global eye health research, while VFC, a global eye health researcher contributed expertise in implementation research and health systems strengthening. Both researchers maintained reflexive awareness of how their professional backgrounds and involvement could influence interpretation. To minimise bias, the evaluation adopted an observational, non-directive approach, and findings were triangulated across quantitative and qualitative data sources, stakeholder groups, and implementation perspectives to enhance the credibility and rigour of the analysis.

## Results

The first implementation cycle screened 9117 patients across multiple districts in Sierra Leone, with 7738 (85%) complete patient records available for analysis. Records with missing or incomplete data were excluded from analyses. A total of 1433 cataract surgeries were performed, representing 72% of the planned annual surgical target. The programme primarily reached middle-aged and older adults (median age 49 years, IQR 35–65) and economically vulnerable populations with 65% (n=5034) earning less than 800 SLL per month (table 3).

**Table 3:** Patient demographics

Patients’ demographics (N=7738)	
	n (%)
Age, years 0–29 30–59 60–89 90 and older N/A Median age (IQR)	n % 1573 (20%) 3305 (43%) 2227 (28%) 32 (1%) 601 (8%) 49 years (IQR 35–65)
Gender	
Male (%) Female (%) N/A (missing) (%)	3228 (42%) 4348 (56%) 162 (2%)
Education	
No formal education	3089 (40%)
Primary school education	868 (11%)
Secondary school education	2140 (28%)
University or higher education	1455 (19%)
N/A (missing)	186 (2%)
Monthly income	
Less than SLL800	5034 (65%)
SLL800–SLL3000	698 (9%)
SLL3000–SLL5000	255 (3%)
Greater than SLL5000	73 (1%)
N/A (missing)	1678 (22%)
Median income (IQR)	400 SLL (0)
Marital status	
Single	1956 (25%)
Married	4184 (54%)
Divorced/separated	186 (2%)
Widowed/widower	1274 (17%)
N/A (missing)	138 (2%)
Eye check history	
Yes	4659 (60%)
No	2740 (35%)
N/A (missing)	339 (5%)
Previous eye surgery	
Yes	533 (7%)
No	6168 (80%)
N/A (missing)	1037 (13%)
District name	
Freetown	5881 (76%)
Port Loko Western area	675 (9%) 1006 (13%)
N/A (missing)	155 (2%)

N/A, not available.

### High demand, access and community engagement

Large patient turnouts across screening and surgical sites highlighted substantial demand for cataract services. Many participants described repeated attempts to access care, early arrival at screening site and long-distance travel, with some receiving formal eye care for the first time

One patient explained:

“Actually, I did not succeed the first day I came because there is always a mad rush whenever Sierra Leoneans hear about free services… Then I came on the third day, and I finally succeeded.”

Transportation costs and multiple travel journeys were common barriers, particularly for rural patients.

Another participant stated:

“Sometimes I had to take three different transports and paid so much money to come here.”

Fear of surgery and misconceptions about blindness also influenced care-seeking. Confidence in surgery increased after observing successful outcomes, receiving family encouragement and reassurance from trusted community and religious leaders

One participant explained:

“I was afraid because I thought they were going to remove all my eyes… but when I saw the first people who were operated on, I developed courage.”

Religious and community leaders played an important role in mobilisation and promoting programme acceptance.

### Delivering high-volume cataract services under real-world constraints

The first implementation cycle operated under substantial operational and infrastructural constraints. High patient turnout placed pressure on patient flow, staffing, documentation and surgical workflow prompting programme teams to expand work shifts, modify staffing arrangements, increase surgical output and adopt high-volume triaging approaches.

Clinicians also reported managing advanced ocular disease and adapting surgical practice to local circumstances

One surgeon highlighted:

“Many patients were postponed because their eye pressures were so uncontrollable and these need to be addressed prior to surgery.”

Another also highlighted:

*“*Ideally, we remove the pterygium first and then plan for the cataract surgery. However, because some patients could not come back, we did both the same day. Which can be a big risk sometimes.”

Poor road infrastructure, long travel distances and seasonal rainfall further disrupted access and postoperative follow-up attendance.

Some patients shared:

“Yes, I am struggling in terms of transportation, because if I do not charter a “keke” [a rickshaw] I won’t be able to come here because, the cars would not pick me because they say I take time to board their cars and it’s a waste of time for them. I can’t tell a lie, I have too many challenges in terms of movement from one place to another.”

Documentation quality was also affected during peak service periods, with missing or incomplete VA data in 46% of records and no VA recorded in 10% of patients (table 4). Limited surgical infrastructure and reliance on visiting ophthalmic teams further constrained programme delivery.

**Table 4:** RE-AIM outcomes from first year cycle

RE-AIM domain	Indicator	Outcome
Reach	Screening coverage Surgical coverage	9117/15 000 (61%) 1433/2000 (72%)
	Gender distribution	Male 42%; female 56%; N/A 2%
	District distribution	Freetown 76%; Port Loko 9%; Western Area 13%; N/A 2%
	Age distribution	<50 years: 47%; ≥50 years: 46%; N/A: 8%
	Severe VI among ≥50 years (VA <3/60)	1686 (48% of ≥50 years)
	Gender distribution among ≥50 years with severe VI	Men 51%; women 49%
Effectiveness	Cataract surgeries completed	1433 successful surgeries
	VA ≥6/12 at 1 week	279 (31%)
	VA <6/12–6/60	532 (59%)
	VA <6/60	89 (10%)
	Absent at 1-week follow-up	533 (37%)
	Surgical complications	No major sight-threatening complications
	Quality of life	+17 mean-point gain (p<0.001)
Adoption		
	Postsurgery follow-up at 1 month	271/1433 (19%)
Implementation	Data completeness	Missing/inaccurate VA: 46%; VA not recorded: 10%
	Screening capacity	Exceeded on multiple days
Maintenance	Care pathway completeness	Limited access to refraction and optical services
	Cost distribution	49% clinical services; 33% patient support
	Infrastructure readiness	One theatre with two operating tables; one slit lamp
	Workforce continuity	Heavy reliance on visiting teams

N/A, not available; RE-AIM, Reach, Effectiveness, Adoption, Implementation and Maintenance; VA, visual acuity; VI, vision impairment.

### Programme effectiveness and patient experience

The programme completed 1433 cataract surgeries during the first implementation cycle. At 1-week follow-up, 31% (n=279) of patients achieved VA of 6/12 or better, while 59% (n=532) had VA between <6/12 and 6/60, and 10% (n=89) had VA worse than 6/60 (table 4). No major sight-threatening surgical complications were reported. Vision-related quality of life improved significantly following surgery, with a mean 17-point increase in composite VFQ-25 scores (p<0.001), particularly in mental health, near vision and distance vision domains (table 5).

**Table 5:** Mean changes in vision-related quality of life subscales following cataract surgery (n=271)

Subscale	Presurgery	Postsurgery	Mean difference	P value
Ocular pain	82.4±24.8	94.1±12.6	11.7	<0.001
Near activity	76.2±32.3	93.3±21.6	17.1	<0.001
Distance activity	82.1±26.8	96.4±15.3	14.3	<0.001
Social functioning	87.5±22.3	98.2±9.8	10.7	<0.001
Mental health	62.7±26.2	92.5±16.5	29.8	<0.001
Role difficulty	83±29.2	95.6±16.0	12.6	<0.001
Dependency	18.8±23.3	22.4±28.5	3.6	0.467
Driving*	54.8±37.6	90.2±14.9	35.4	1
Peripheral vision	86.7±26.1	99.2±6.8	12.5	<0.001
Composite score	70.5±4.2	87.5±6.3	17	<0.001

Scoring was not normally distributed, hence non-parametric analysis was done. VFQ-25 composite and subscale scores were calculated according to the NEI VFQ-25 scoring algorithm, using mean values of completed items transformed to a 0–100 scale. Shapiro-Wilk tests indicated non-normal distributions for most scores (p<0.05). Therefore, non-parametric Wilcoxon signed-rank tests were used to compare presurgery and postsurgery scores.

* Driving (n=17) scores were based on a limited number of respondents due to low completion rates.

NEI VFQ-25, National Eye Institute Visual Function Questionnaire.

One patient stated:

“After my surgery, when my eyes healed, I could finally see how beautiful my house looked.”

Clinicians also described visible changes in patients’ mood and behaviour following surgery.

“Patients were dancing and singing the next day because they could see again after 20–30 years.”

Follow-up attendance declined from 63% (900/1433) at 1 week to 19% (271/1433) at 1 month. Transportation costs, dependence on caregivers, poor road conditions and seasonal rainfall were consistently identified as major barriers to postoperative follow-up, particularly among older adults.

One participant stated:

“My son who would bring me to see the doctors went to work that day and if he is not around to send me, no one will. As you can see, I am old and weak and can’t travel alone.”

Seasonal conditions and road safety concerns during the rainy season were also frequently reported.

“Transportation in Sierra Leone is a big problem for us especially during the raining season, it can rain from morning to evening and this make the road very bad and unsafe.”

No significant associations were observed between demographic characteristics and changes in composite vision-related quality-of-life scores (online supplemental table S1). In subscale-specific analyses, sex was significantly associated with changes in peripheral vision scores (β=0.254, p=0.047), while no other predictors reached statistical significance (online supplemental table S2).

### Programme learning and adaptation for future cycles

Experiences from the first implementation cycle informed several proposed operational adaptations for subsequent programme cycles. Programme implementers identified the need for revised screening targets and more structured patient flow management during peak service periods. Proposed modifications included introduction of daily screening caps, expansion of staffing during anticipated high-demand periods, and assignment of dedicated data personnel, separate from clinical teams and recording of first day postoperative VA.

Programme teams additionally proposed greater decentralisation of postoperative follow-up services through district hospitals to reduce transportation burden for patients residing outside Freetown. Other proposed adaptations included strengthened referral tracking systems, increased use of phone reminders, digitisation of patient records, additional ophthalmic infrastructure investment and structured pre-camp training for local staff. Seasonal scheduling adjustments, particularly increased alignment of outreach activities with dry-season periods, were also identified as priorities for future implementation cycles. Postoperative complications were not systematically recorded within the routine programme dataset during the first implementation cycle, preventing reliable assessment of complication rates or patterns. This was identified as a gap in programme monitoring and postoperative outcome documentation (figure 3).

**Figure 3:**
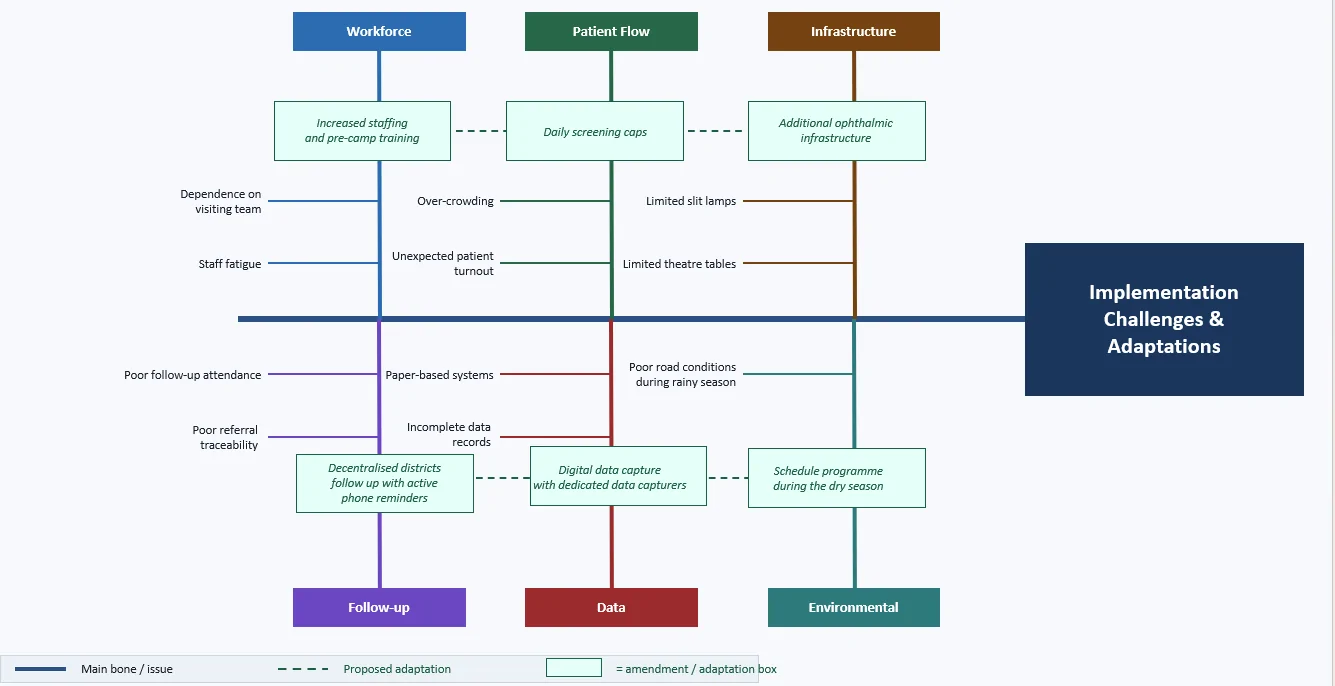
Implementation challenges and adaptations during programme delivery. Fishbone diagram illustrating the main implementation challenges identified across six domains: workforce, patient flow, infrastructure, follow-up, data and environmental factors. Challenges encountered during implementation are shown along each domain, while the green boxes indicate adaptations introduced or proposed in response, including increased staffing and pre-camp training, daily screening caps, additional ophthalmic infrastructure, decentralised follow-up with active phone reminders, digital data capture with dedicated data personnel and scheduling activities during the dry season. Dashed lines indicate the relationship between identified challenges and corresponding adaptations.

## Discussion

The study assessed the establishment and operationalisation of a high-volume cataract outreach programme within a fragile and resource-constrained setting While the programme achieved substantial surgical output and reached vulnerable populations across several districts, the findings highlight the complexity of establishing high-volume cataract outreach services within a resource-constrained setting. Rather than operating as a fixed intervention, the programme evolved continuously in response to patient demand, workforce pressures, infrastructural limitations and barriers affecting continuity of care. These experiences provide important implementation learning for strengthening future outreach cycles and similar cataract service delivery models in comparable low-resource settings.

Importantly, many of the operational modifications introduced during cycle 1 emerged iteratively during implementation rather than through predefined planning processes alone. Changes to staffing arrangements, patient flow systems, scheduling approaches, follow-up coordination and documentation processes reflected adaptive responses to emerging implementation realities encountered during programme delivery. This reflects the value of viewing outreach programmes as evolving service-delivery systems that require continuous adjustment and contextual responsiveness rather than rigid implementation models.

### Demand, access and hidden unmet need

The first implementation cycle revealed a substantial unmet need for cataract surgery and eyecare services in Sierra Leone. High patient turnout, repeated attempts to access care and long-distance travel indicated that the burden of untreated cataract exceeded initial programme assumptions. Many participants reported accessing cataract surgery for the first time despite years of visual impairment. These findings are consistent with evidence from sub-Saharan Africa, where delayed cataract service utilisation is driven by geographic barriers, poverty, limited surgical capacity and weak referral systems rather than unwillingness to seek care.^16^ Similar reports from Kenya, Uganda and Zambia highlight significant hidden cataract backlogs and poor continuity of care.^16^ Qualitative findings further demonstrated that fear of surgery and misconceptions about blindness influenced treatment uptake, while trust in successful patient outcomes, family support and community and religious leaders promoted acceptance. These findings align with previous studies emphasising the importance of culturally responsive community engagement in improving cataract service uptake across sub-Saharan Africa.^17 18^

### Delivering high-volume services under operational constraint

A major finding was the operational complexity of delivering high-volume cataract outreach within a resource-constrained health system. Higher-than-expected screening and surgical volumes placed pressure on staffing, patient flow, documentation and surgical delivery, requiring adaptive measures including expanded work shifts, revised staffing, high-volume triage and workflow redesign. These findings highlight the challenge of balancing rapid service delivery with documentation quality, patient monitoring, continuity of care and workforce sustainability. Similar operational constraints have been reported in cataract outreach programmes across sub-Saharan Africa.^8^ The programme also exposed broader health system limitations, including reliance on visiting ophthalmic teams, limited surgical infrastructure and centralised tertiary care. Overall, the findings demonstrate that high-volume cataract outreach in low-resource settings requires flexible, adaptive implementation models capable of responding to changing service demands and contextual pressures.

### Follow-up and continuity of care as structural issues

Another important finding relates to postoperative follow-up attendance. Although follow-up decline is often discussed within outreach programmes as a problem of patient non-compliance, findings from this study suggest that continuity of care was strongly shaped by structural and systems-level barriers. Transportation costs, dependence on caregivers, poor road infrastructure, long travel distances and seasonal rainfall disruptions all influenced patients’ ability to attend postoperative review appointments. Although, 30% of patients achieved postoperative VA better than 6/12 at week one. This finding should be interpreted cautiously because VA was assessed during the early postoperative phase and may not represent the final visual outcome. Nevertheless, 10% of patients had VA <6/60 1 week postoperative. The underlying causes of poor postoperative VA were not systematically collected in the routine dataset, limiting our ability to determine whether these outcomes were attributed to pre-existing ocular comorbidity, surgical factors or other factors.

For many older patients, attendance depended not only on personal willingness, but also on household support, caregiver availability, transportation access and financial capacity. Similar findings have been reported in other low-resource settings where postoperative attrition reflects broader infrastructural and socioeconomic constraints rather than behavioural unwillingness alone.^8 19^ Conceptually, these findings suggest that follow-up attendance within outreach programmes should be understood as a health systems accessibility issue rather than solely an issue of individual patient responsibility.

The findings therefore highlight the importance of decentralised follow-up systems, strengthened referral tracking pathways, district-level postoperative review mechanisms and transportation support strategies for improving continuity of care within cataract outreach programmes operating across geographically dispersed settings.

### Patient experience, trust and programme acceptability

Despite operational and follow-up challenges, cataract surgery resulted in marked improvements in patients’ daily functioning, independence, mobility and social participation. Significant gains in VFQ-25 scores, particularly in mental health, near vision and social functioning, highlight the broader psychosocial and functional benefits of surgery beyond improvements in VA alone. Although 1-week postoperative visual outcomes remained below WHO recommended thresholds,^20^ these findings should be interpreted within the context of advanced cataract presentations, coexisting ocular conditions, delayed treatment seeking and poor 1-month follow-up attendance, which limited assessment of longer-term recovery.

Importantly, the findings demonstrate that programme acceptability was shaped not only by clinical outcomes, but also by trust in programme personnel and confidence in the perceived expertise of visiting surgical teams.^17 18^ Visible improvements among treated patients strengthened community confidence, consistent with evidence from other low-resource settings demonstrating that cataract surgery enhances quality of life and social participation beyond restoring vision.^21–23^

### Sustainability, adaptation and implications for future cycles

The first implementation cycle highlighted important sustainability challenges within the outreach model. Although the programme achieved high surgical output, service delivery remained dependent on visiting ophthalmic teams, limited infrastructure and centralised tertiary care, raising questions about long-term workforce capacity, decentralisation and integration within the national eye-care system. Many proposed improvements emerged directly from implementation experience, including strengthened patient selection, enhanced postoperative assessment, decentralised follow-up, improved district coordination, dedicated data personnel, revised scheduling, local workforce training, digitised patient records, improved referral tracking and greater alignment of outreach activities with the dry season. These findings demonstrate that high-volume cataract outreach should be viewed as an evolving service-delivery system rather than a fixed intervention.

The first cycle also identified limitations in the programme’s ability to monitor surgical quality beyond early VA outcomes. Postoperative complications and their causes were not systematically captured, limiting assessment of surgical safety and interpretation of suboptimal postoperative vision. Importantly, this represents not only a limitation of the present evaluation but also an implementation learning point: strengthening standardised documentation of surgical complications and postoperative outcomes should form part of the programme’s quality-assurance system in subsequent cycles.

### Strengths and limitations

A key strength of this study was the combined use of the RE-AIM framework and PDSA approach, which provided a comprehensive structure for evaluating programme implementation while simultaneously capturing iterative operational adaptations during service delivery. This combination enabled assessment not only of programme reach and effectiveness, but also of how implementation processes evolved in response to real-world contextual pressures.

Despite these contributions, several limitations should be considered when interpreting the findings from this study. First, reliance on routine programme data resulted in some missing and incomplete records. Second, multiple operational adaptations occurred concurrently throughout implementation, making it difficult to isolate the individual effects of specific modifications on programme outcomes. Visual outcomes were assessed only at 1-week postoperative follow-up, therefore, these findings may not reflect final visual outcomes, which may continue to improve. The routine dataset did not systematically capture causes of suboptimal postoperative VA, limiting interpretation of the early postoperative visual outcomes. Finally, the evaluation focused primarily on the first implementation cycle and was therefore unable to assess longer-term sustainability, maintenance and health-system integration outcomes over time.

## Conclusions

Taken together, these findings demonstrate that the first implementation cycle functioned as an adaptive learning phase that generated critical operational insights for future programme refinement. The programme evolved continuously throughout implementation in response to workforce strain, documentation challenges, patient flow pressures, transportation barriers and follow-up limitations. Importantly, many of the adaptations proposed following cycle 1—including improved patient selection criteria and first day postoperative care, decentralised follow-up systems, strengthened district-level coordination, dedicated data personnel, revised scheduling strategies and increased workforce preparation—emerged directly from implementation experiences encountered during programme delivery. This highlights the value of viewing cataract outreach programmes not as static interventions, but as evolving service delivery ecosystems requiring continuous adaptation, systems learning and contextual responsiveness. In this regard, the combined use of RE-AIM and PDSA frameworks provided a useful structure not only for evaluating programme outcomes, but also for understanding how implementation realities shaped programme evolution during the establishment phase of a large-scale cataract outreach initiative in Sierra Leone.

## Supplementary material

10.1136/bmjopen-2026-126880 online supplemental file 1

## Data Availability

Data are available on reasonable request.
