# Supplementary material for: What are the implementation lessons from a high-volume cataract screening and surgical programme? A mixed-methods implementation evaluation in Sierra Leone
Source: BMJ Open. 2026 Oct 9;16(10):e126880. doi: 10.1136/bmjopen-2026-126880 (PMC13656124; doi:10.1136/bmjopen-2026-126880)
Supplement: Supplementary file 1 [file bmjopen-2026-126880.reviewer_comments.pdf]

PEER REVIEW HISTORY

BMJ Open publishes all reviews undertaken for accepted manuscripts. Reviewers are asked to complete a checklist review form and are provided with free text boxes to elaborate on their assessment. These free text comments are reproduced below.

ARTICLE DETAILS

Title (Provisional)

What are the implementation lessons from a high-volume cataract screening and surgical programme? A mixed-methods implementation evaluation in Sierra Leone

Authors

Osei-Anokye, Gabriel; Rabiu, Muhammad; Harrison-Williams, Lloyd; Mattia, John G; Chan, Ving Fai

---

VERSION 1 - REVIEW

**Reviewer**	**1**
**Name**	**Bowen, Michael**
**Affiliation**	**The College of Optometrists, Research**
**Date**	**18-Aug-2026**
**COI**	**None**

It would be helpful to cite a checklist for the qualitative elements of the study - SRQR or similar.

Running through the SRQR checklist will highlight that some additional details are needed - there is currently no clear definition of the research paradigm and although the qualitative approach has been described briefly, it would helpful to relate the described approach to established qualitative approaches. It is positive to see a clear reflexivity statement included - thank you.

IN the Qualitative analysis section (243 - 256) it would be helpful to make clear which researchers reviewed the qualitative data and whether / how any differences in their review of those data were dealt with.

The discussion section would lead the reader to infer that a more complete thematic analysis may have been undertaken, and that the section headings might relate to themes that emerged, however, there is not enough information to allow the reader to really understand the outcomes (or to aim to replicate the process) from the "framework-informed interpretive approach" that is referred to in the Qualitative analysis section. It might be

helpful to provide a process diagram to clarify this to readers and to support any future work that aimed to replicate this research.

**Reviewer**	**2**
**Name**	**Biswas, Kaberi**
**Affiliation**	**SNIMS, Ophthalmology**
**Date**	**28-Aug-2026**
**COI**	**None**

The write up is interesting and relevant.

1. The abstract and introduction could be shortened and made more crisp. The abstract seems very long. The results and conclusions could be summarised in a couple of sentences in the abstarct. The RE AIM and PDSA could be explained in the introduction itself. The drawbacks faced by patients in Sierra Leone could be explained in a couple of sentences instead of 2 paragraphs.

2. Many abbreviations like GOA,VFC and VA have not been explained. After the abstract, a list of abbreviations with the full form could be placed.

3.Only about 30% of the patients had visual acuity better than 6/12 postoperatively. The causes of this could be explained. No further details were given. Also the causes of the poor visual acuity in 10% of patients needs more explanation since this is extremely relevant to the program. the poor follow up attendance is also a very important concern and needs looking into.

4. Some of the references are not complete and references as old as 2014 have been included.

VERSION 1 - AUTHOR RESPONSE

Reviewer 1:

Comment: It would be helpful to cite a checklist for the qualitative elements of the study - SRQR or similar.

Response: Thank you for this helpful suggestion. We agree that reporting the qualitative component in accordance with an established reporting guideline will improve the transparency and completeness of the study. We have therefore used the SRQR checklist to guide the reporting of the qualitative elements of the study and have added the appropriate citation to the manuscript. The completed SRQR checklist has also been provided as a supplementary file.

Comment: Running through the SRQR checklist will highlight that some additional details are needed - there is currently no clear definition of the research paradigm and although the qualitative approach has been described briefly, it would helpful to relate the described approach to established qualitative approaches. It is positive to see a clear reflexivity statement included - thank you.

Response: Thank you for this constructive feedback and for acknowledging the reflexivity statement. The manuscript has been revised based on the SRQR checklist.

Comment: IN the Qualitative analysis section (243 - 256) it would be helpful to make clear which researchers reviewed the qualitative data and whether / how any differences in their review of those data were dealt with.

Response: Thank you for this helpful comment, we have revised the qualitative analysis section to clarify the researchers involved in reviewing and analysing the qualitative data. Two researchers (GOA and VFC) reviewed the interview and focus group transcripts. Their interpretation of the data were compared, and any differences in coding or interpretation were discussed between the two researchers and resolved through consensus.

Comment: The discussion section would lead the reader to infer that a more complete thematic analysis may have been undertaken, and that the section headings might relate to themes that emerged, however, there is not enough information to allow the reader to really understand the outcomes (or to aim to replicate the process) from the "framework-informed interpretive approach" that is referred to in the Qualitative analysis section. It might be helpful to provide a process diagram to clarify this to readers and to support any future work that aimed to replicate this research.

Response: Thank you. We agree that the previous description did not provide sufficient detail for readers to understand how the framework-informed qualitative analysis was undertaken. We have therefore expanded the Qualitative Analysis subsection to describe the analytical process, including familiarisation, identification and organisation of implementation-relevant data, use of the RE-AIM domains and implementation-learning objectives, comparison across stakeholder groups, consensus between the two researchers, and integration with quantitative findings. We have also added a process diagram illustrating the analytical pathway. We have clarified that this was a qualitative descriptive, framework-informed interpretive analysis rather than a standalone thematic analysis.

Reviewer 2

Comment: The abstract and introduction could be shortened and made more crisp. The abstract seems very long. The results and conclusions could be summarised in a

couple of sentences in the abstract. The RE AIM and PDSA could be explained in the introduction itself. The drawbacks faced by patients in Sierra Leone could be explained in a couple of sentences instead of 2 paragraphs

Response: Thank you for this clarification. We have retained only a brief reference to RE-AIM and PDSA in the Introduction and moved the detailed description of both frameworks to the Conceptual Framework subsection of the Methods.

Comment: Many abbreviations like GOA,VFC and VA have not been explained. After the abstract, a list of abbreviations with the full form could be placed.

Response: Thank you for this helpful comment. We agree that several abbreviations were not clearly defined for readers. We have reviewed the manuscript and ensured that all abbreviations are defined at their first occurrence in the main text. Two researchers (GOA and VFC) independently reviewed interviews and focused group discussion transcripts.

Comment: Only about 30% of the patients had visual acuity better than 6/12 postoperatively. The causes of this could be explained. No further details were given. Also the causes of the poor visual acuity in 10% of patients needs more explanation since this is extremely relevant to the program. the poor follow up attendance is also a very important concern and needs looking into.

Response: Thank you for highlighting the importance of the postoperative visual acuity findings. We have clarified these results in the revised manuscript. At one-week postoperative follow-up 31% of patients had VA ≥6/12, 59% had VA between <6/12 and 6/60, and 10% had VA <6/60. We recognise that these measurements were obtained at one week, whereas WHO cataract surgical visual outcome categories are assessed at four to six weeks postoperatively. We have therefore removed the direct comparison of our one-week outcomes with the WHO outcome categories and now describe these findings as early postoperative visual outcomes.

We also agree that understanding the causes of poor postoperative visual acuity is important for programme quality assurance. The routine dataset used during the first implementation cycle did not systematically capture postoperative complications or the clinical causes of suboptimal postoperative visual acuity. We are therefore unable to determine the causes of VA <6/60 reliably and have avoided attributing these outcomes to specific clinical or surgical factors. We have clarified this in the Results and Limitations. Importantly, we have also identified the absence of systematic complication and postoperative outcome recording as an implementation finding from the first cycle and discuss the need to strengthen these monitoring processes in subsequent programme cycles.

Comment: Some of the references are not complete and references as old as 2014 have been included.

Response: Thank you for highlighting this. We have carefully reviewed and revised the reference list to ensure that the bibliographic details are complete. We have also updated selected references where more recent and relevant evidence was available. Some older references have been retained where they provide important evidence that remains directly relevant to the study.
