# Supplementary material for: What are the implementation lessons from a high-volume cataract screening and surgical programme? A mixed-methods implementation evaluation in Sierra Leone
Source: BMJ Open. 2026 Oct 9;16(10):e126880. doi: 10.1136/bmjopen-2026-126880 (PMC13656124; doi:10.1136/bmjopen-2026-126880)
Supplement: Supplementary file 2 [file bmjopen-16-10-s001.docx]

***Supplementary Table 1: Demographic Factors Associated with Changes in Vision-Related Quality-of-Life Scores Following Cataract Surgery***

| **Variable** | **Test** | **Statistic** | **p-Value** |
| --- | --- | --- | --- |
| Age | Spearman correlation | ρ= 0.148 | 0.062 |
| Gender | Wilcoxon rank-sum | W = 3389 | 0.640 |
| Education | Kruskal-wallis | X^2^ = 1.37 | 0.850 |
| Income | Kruskal-wallis | X^2^ = 4.41 | 0.220 |
| Marital status | Kruskal-wallis | X^2^ = 5.86 | 0.120 |

*Footnote****:*** *Changes in vision-related quality-of-life scores across different demographic variable of patients. Spearman's rank correlation was used to assess associations with age, the Wilcoxon rank-sum test was used for binary variables (gender), and the Kruskal–Wallis test was used for categorical variables with more than two levels (education, income, and marital status). Statistical significance was defined as p < 0.05.*

**Supplementary Table 2. Multivariable regression analysis of demographic and clinical factors associated with changes in vision-related quality-of-life subscale scores following cataract surgery.**

| **Outcome (subscale)** | **Adjusted R^2^** | **P-value** |
| --- | --- | --- |
| Ocular pain | 0.030 | 0.322 |
| Near Activity | -0.173 | 0.998 |
| Distance Activity | -0.140 | 0.986 |
| Social functioning | -0.153 | 0.992 |
| Mental Health | -0.135 | 0.983 |
| Role difficulty | -0.155 | 0.994 |
| Dependency | -0.104 | 0.935 |
| Peripheral Vision | **0.163** | **0.025** |

*Footnote: Multiple linear regression models were fitted for each vision-related quality-of-life subscale using change scores as the dependent variables. All models were adjusted for age, sex, marital status, education, income, district of residence, family history of cataract, and previous ocular surgery. Statistical significance was defined as p < 0.05.*

**Supplementary table legends**

Supplementary table 1: *Association between demographic characteristics and changes in vision-related quality-of-life scores following cataract surgery. Statistical tests were selected according to the distribution and measurement level of each demographic variable. Statistical significance was defined as* *p* < 0.05. *ρ, Spearman's rank correlation coefficient; W, Wilcoxon rank-sum test statistic; χ², Kruskal–Wallis test statistic.*

Supplementary table 2: *Multivariable linear regression models examining the association of demographic and clinical factors with changes in individual vision-related quality-of-life subscale scores following cataract surgery. Adjusted R² represents the proportion of variance in each subscale change score explained by the variables included in the model, adjusted for the number of predictors. Statistical significance was defined as* *p*<0.05.
